# Controlling weeds with fungi, bacteria and viruses: a review

**DOI:** 10.3389/fpls.2015.00659

**Published:** 2015-08-28

**Authors:** Dylan P. Harding, Manish N. Raizada

**Affiliations:** Department of Plant Agriculture, University of Guelph, Guelph, ON, Canada

**Keywords:** bioherbicide, herbicide resistance, turf, *Colletotrichum*, *Phoma*, *Sclerotinia*, *Xanthomonas*, *Pseudomonas*

## Abstract

Weeds are a nuisance in a variety of land uses. The increasing prevalence of both herbicide resistant weeds and bans on cosmetic pesticide use has created a strong impetus to develop novel strategies for controlling weeds. The application of bacteria, fungi and viruses to achieving this goal has received increasingly great attention over the last three decades. Proposed benefits to this strategy include reduced environmental impact, increased target specificity, reduced development costs compared to conventional herbicides and the identification of novel herbicidal mechanisms. This review focuses on examples from North America. Among fungi, the prominent genera to receive attention as bioherbicide candidates include *Colletotrichum*, *Phoma*, and *Sclerotinia*. Among bacteria, *Xanthomonas* and *Pseudomonas* share this distinction. The available reports on the application of viruses to controlling weeds are also reviewed. Focus is given to the phytotoxic mechanisms associated with bioherbicide candidates. Achieving consistent suppression of weeds in field conditions is a common challenge to this control strategy, as the efficacy of a bioherbicide candidate is generally more sensitive to environmental variation than a conventional herbicide. Common themes and lessons emerging from the available literature in regard to this challenge are presented. Additionally, future directions for this crop protection strategy are suggested.

## Recent History of Weed Control

Weeds are a problem in both crop production and turfgrass systems, associated with declines in crop yields and quality, as an esthetic nuisance and as a source of allergenic pollen (Stewart-Wade et al., 2002; Oerke, 2006; Gadermaier et al., 2014). Since the post-World War II introduction of the first selective herbicides, 2,4-D and MCPA, such products have significantly changed the management techniques that are employed by farmers and other managers of anthropogenic ecosystems (Mithila et al., 2011). The primary benefit offered by selective herbicides is the ability to control certain weed species without harming crops, based on physiological differences between species. This ability has enabled significant yield increases in many crops, and continues to be an important aspect of agroecosystem management (Mithila et al., 2011). Currently, there are 25 known herbicide target sites at the molecular level (Heap, 2015), e.g., disruption of EPSP synthase required for branched amino acid synthesis by glyphosate (Sammons and Gaines, 2014) or interference of auxin pathways by 2,4-D (Grossmann, 2010). Despite this variety, in many cases a limited number of herbicide mechanisms have been continuously employed by operators based on the low cost or ease of use associated with those products (Beckie, 2011; Mithila et al., 2011). This practice has in many cases created artificial selection pressure on weed populations, causing the widespread emergence of herbicide-resistant weeds (Beckie et al., 1999; Green and Owen, 2011; Mithila et al., 2011; Darmency, 2013). As of June, 2015, resistant-weed populations have been reported in association with 22 of the 25 known herbicide targets (Heap, 2015).

The introduction of glyphosate-resistant transgenic crops brought a novel strategy for controlling weeds to the array of options available to operators (Green and Owen, 2011). This approach was notably different from previous selective weed control options in that it enabled the application of a broad spectrum herbicide that controlled almost all plants that were not engineered to tolerate glyphosate (Green and Owen, 2011). The unprecedented efficacy and ease of use associated with this weed control system led to its rapid adoption throughout much of the world, in many cases completely replacing former weed control practices (Beckie, 2011; Green and Owen, 2011). However, as with the generation of selective herbicides before it, the common practice of continuous glyphosate use led to the emergence of resistant weed populations, beginning in 1996 with the observation of glyphosate-resistant rigid ryegrass (*Lolium rigidum*) in Australia (Green and Owen, 2011). As of the writing of this report, there are 32 glyphosate-resistant weed species throughout the world (Heap, 2015).

It is apparent that as new herbicides are developed, weeds will continue to evolve in response to whatever selective pressure that is applied. For this reason, the continuous development of novel weed control methods is essential to the ongoing maintenance of agricultural yields. These developments are needed both to control weed populations that are resistant to currently available modes of action, as well as to diversify weed control platforms in order to delay the emergence of new resistance traits. Additionally, increasing public concern with the negative effects of pesticide residues, particularly in residential areas (e.g., turfgrass), has led to increasing demand for alternative methods of controlling weeds and other pests (Knopper and Lean, 2004; Bailey et al., 2010; Belair et al., 2010).

## Biological Control of Weeds: Introduction and Scope of Review

Biological control as a general term refers to the introduction of organisms into an ecosystem with the intention of controlling one or more undesirable species (Charudattan, 2001; Bailey et al., 2010). Within the context of controlling weeds and otherwise invasive plant species, this field of study has increasingly focused on bacteria and fungi in the past five decades (Li et al., 2003), although viruses have also been considered for this purpose in select cases (Ferrell et al., 2008; Elliott et al., 2009; Diaz et al., 2014).

There are two primary fields of application within the study of biological weed control. Classical biological control refers to the release of a natural predator or pathogen of a pest species with the anticipation that it will be able to persist in the environment and provide ongoing reduction of the pest species population throughout an entire ecosystem (Dane and Shaw, 1996; TeBeest, 1996; Shaw et al., 2009). On the other hand, inundative biological control (also referred to as the bioherbicide strategy) refers to the application of propagation materials such as fungal spores or bacterial suspensions in concentrations that would not normally occur in nature, with the intention of destroying a pest species within a managed area (Johnson et al., 1996; TeBeest, 1996). The inundative biological control strategy is more relevant to the needs of agriculture and turf management, as it can generally be implemented through the application of inoculum as liquid sprays or solid granules in a similar manner to conventional herbicides (Auld et al., 2003; Caldwell et al., 2012).

In Canada, a significant number of biological agents for control of insects, plant pathogens and weeds has been approved by the Pest Management Regulatory Agency (PMRA), with 24 such products registered between 1972 and 2008, and the majority of these registrations occurring between 2000 and 2008 (Bailey et al., 2010). An even greater number of microbes and microbe-derived chemicals has been registered with the United States Environmental Protection Agency (EPA) for crop, forest, or ecological management, with 53 such products registered between 1996 and 2010 (EPA, 2014). As of 2014, 47 different microbial strains were approved in the EU for the purpose of controlling fungi or insects (European Parliament, 2014). Surprisingly, there are no microbes approved for the control of weed species in the European Union (European Parliament, 2014).

This review will focus on the use of biological agents to control weeds, including fungi, bacteria and viruses, with examples provided from North America. The review will discuss incentives to adopt these technologies, factors in the real world that affect their efficacy, and challenges to their commercialization. The review will conclude by examining future directions to accelerate progress in this promising field.

### Incentives to Adopt Biological Agents to Control Weeds

The use of bioherbicides in lieu of traditional chemical inputs has the potential to offer a number of benefits to managers of ecological systems, pesticide producers and the general public. Most proponents of biological control strategies cite reduced environmental impact as the primary benefit associated with such management techniques (Auld and Morin, 1995; Johnson et al., 1996; Li et al., 2003; Ghosheh, 2005). This argument has been put forth on the basis of increased target specificity (Auld and Morin, 1995), the rapid degradation of residual biological weed control agent metabolites (Li et al., 2003), and the inability of bioherbicide species to propagate without human assistance (Johnson et al., 1996; Hoagland et al., 2007). It has also been argued that the unintended dispersal of introduced biological weed control species can be limited through the employment of agents that cannot survive without their particular host, such as certain strains of *Xanthomonas* (Schaad et al., 2001). The development cost associated with bioherbicides has also been reported to be generally lower than the cost of developing a comparable chemical agent (Auld and Morin, 1995; Li et al., 2003). Finally, as the public perception of pesticides is generally negative, the development and implementation of lower-risk pest control strategies has the potential to capture the increased willingness of consumers to pay premium prices for foods produced through these methods (Anderson et al., 1996; Bazoche et al., 2014). This has been specifically investigated by McNeil et al. (2010) through a telephone survey of Canadian consumers, in which 70% of participants indicated preference for foods produced using biological control agents rather than synthetic insecticides. It is likely that a similar trend would emerge in regard to consumer preference for biological herbicides over conventional herbicides.

The pressure to develop novel weed control strategies has been additionally increased by the removal of several effective but environmentally problematic pesticides from various markets (Charudattan, 2001). Biological weed control strategies can potentially address this need and provide novel modes of action that will inhibit the growth of weeds that are resistant to more commonly used herbicides. Additionally, it is also possible that in some cases biological control agents could be applied in combination with herbicides to attack weed species through multiple modes of action (Auld and Morin, 1995; TeBeest, 1996).

## Biological Control of Weeds Using Fungi

A list of the biological weed control candidates described in this article is summarized (Table 1). Most commercial biological weed control products researched in North America have been based on formulations of fungal species, however, few have been successful in the long term. Examples include BioMal, a formulation of *Colletotrichum gloeosporioides* f.sp. *malvae*, introduced for the control of round leaf mallow (*Malva pusilla*) (Mortensen, 1988; PMRA, 2006), and *C. gloeosporioides* f.sp. *aeschynomene*, which was released for control of northern jointvetch (*Aeschynomene virginica*) in the United States in 1982 as Collego (Daniel et al., 1973; Menaria, 2007), and again in 2006 as LockDown (EPA Registration Number 82681-1) (Bailey, 2014). Additionally, Sarritor, a formulation of *Sclerotinia minor* was introduced for the control of dandelion (*Taraxacum officinale*), white clover (*Trifolium repens*) and broadleaf plantain (*Plantago major*) in turf (PMRA, 2010).

**TABLE 1:** **Summary of North American biocontrol agents discussed**.

**Bioherbicide agent**	**Target weed**	**Specificity of control agent**	**Intended system**	**Product formulation**	**Stage of development**	**Initial scientific report**
**Fungal agents**						
*Colletotrichum gloeosporioides* f.sp. *aeschynomene*	Northern jointvetch (*Aeschynomene virginica*)	Also affects *Sesbania exaltata* Boyette et al. (2011)	Field crops: Rice, soybean	Aqueous spore suspensions Sandrin et al. (2003)	Registered with EPA in 1982, no longer available	Daniel et al. (1973)
*Colletotrichum gloeosporioides* f.sp. *malvae*	Round leaf mallow (*Malva pusilla*)	Lethal effect limited to *Malvaceae* family Mortensen (1988)	Field crops: Wheat, rye, flax, lentil, barley, canola, sunflower, soybean, oats, mustard, sugar beet and buckwheat	Aqueous spore suspension Mortensen (1988)	Registered with PMRA from 1992 to 1994 (marketed by Philom Bios Inc.)	Mortensen (1988)
*Colletotrichum orbiculare*	Spiny cocklebur (*Xanthium spinosum*)	Known pathogen of the *Cucurbitaceae* Harata and Kubo (2014)	Pasture and field crops	Aqueous spore suspension Auld et al. (1988)	Research phase	Auld et al. (1988)
*Colletotrichum truncatum*	Hemp sesbania (*Sesbania exaltata*)	Pathogenicity reported as limited to *Leguminosae* Boyette (1991) Minor pathogenicity on *Matricaria perforata* Hynes et al. (2010)	Field crops	Aqueous spore suspension Boyette et al. (2007)	Research phase	Boyette (1991)
*Phoma chenopodicola*	Lamb’s quarters (*Chenopodium album*), Creeping thistle (*Cirsium arvense*), Green foxtail (*Setaria viridis*), Annual mercury (*Mercurialis annua*)	Not tested on other species	Field crops such as sugar beet and corn	Active ingredient isolated from live culture of *P*. *chenopodicola* using organic solvents [MeOH-H_2_O (1:3) solution] Cimmino et al. (2013)	Research phase	Cimmino et al. (2013)
*Phoma herbarum*	Dandelion (*Taraxacum officinale*)	Reported as a potential control agent for *Trianthema portulacastrum* Ray and Vijayachandran (2013)	Turf	Suspension of mycelia in potato dextrose broth Neumann and Boland (1999)	Research phase	Neumann Brebaum (1998); Neumann and Boland (1999)
*Phoma macrostoma*	Dicot plants	Affects most monocots but not dicots Bailey et al. (2011)	Turf	Granules composed of mycelial fragments and flour (final iteration of inoculum formula) Bailey et al. (2011)	94-44B strain registered with EPA and PMRA in 2011	Graupner et al. (2003); Bailey et al. (2011)
*Sclerotinia minor*	Dandelion (*Taraxacum officinale*), White clover (*Trifolium repens*), and Broadleaf plantain (*Plantago minor*)	Wide host range, predominantly dicot species Melzer et al. (1997)	Turf	Barley grits containing actively growing mycelia Abu-Dieyeh and Watson (2006)	Registered with PMRA as Sarritor in 2010, no longer available	Riddle et al. (1991)
*Chondrostereum purpureum* strain HQ1	Re-growth of deciduous trees and shrubs	Wide host range Setliff (2002)	Forestry	Paste containing mycelia PMRA (2002)	Registered as Mycotech Paste with PMRA in 2002, and with EPA in 2005	Setliff (2002)
*Chondrostereum purpureum* strain PFC 2139	Re-growth of deciduous trees and shrubs	Wide host range Setliff (2002)	Forestry	Paste containing mycelia EPA (2004)	Registered as Chontrol Paste with EPA in 2004, and with PMRA in 2007, commercially available in both regions	Setliff (2002)
*Puccinia thlaspeos*	Dyer’s woad (*Isatis tinctoria*)	*Isatis tinctoria* only EPA (2002)	Ecological management	Spray containing leaf fragments of infected *Isatis tinctoria*	Registered as Woad Warrior with EPA in 2002, no longer commercially available	Lovic et al. (1988)
*Alternaria destruens*	Dodder species (*Cuscata* spp.)	Observed to affect several unspecified crop species EPA (2005)	Alfalfa, cranberries, carrots, peppers, tomatoes, eggplant, blueberries, and woody ornamentals	Wettable power and granules containing spores	Registered as Smolder G and Smolder WP with EPA in 2005, no longer commercially available	Simmons (1998)
*Phytophthora palmivora*	Strangler vine (*Morrenia odorata*)	Weak pathogen of some crop species Ridings et al. (1973)	Citrus orchards	Aqueous spore suspension	Registered as DeVine with EPA in 1981, re-registered in 2006. No longer commercially available	Burnett et al. (1973)
**Bacterial agents**						
*Pseudomonas fluorescens* strain D7	Downy brome (*Bromus tectorum*)	Effect is limited to *Bromus tectorum* Kennedy et al. (2001)	Field crops	Cell-free filtrate prepared from nutrient broth culture Gealy et al. (1996)	Research phase	Kennedy et al. (1991)
*Pseudomonas fluorescens* strain BRG100	Green foxtail (*Setaria viridis*)	Not identified	Not specified	Granules prepared from live culture in nutrient broth, oat flour, maltose and molasses Caldwell et al. (2012)	Research phase	Quail et al. (2002)
*Pseudomonas fluorescens* strain WH6	Inhibits most of the species tested	Non-specific	Not specified	Cell-free filtrate Banowetz et al. (2008)	Research phase	Banowetz et al. (2008)
**Viral agents**						
Tobacco Mild Green Mosaic Tobamovirus	Tropical soda apple (*Solanum viarum*)	Also affects *Capsicum* spp. and *Nicotiana* spp. Font et al. (2009); EPA (2015)	Pastures	Extract from virus-infected tobacco leaf in sodium phosphate buffer used in experimental phase	Registered with EPA in 2015	Ferrell et al. (2008)
*Araujia* Mosaic Virus	Moth plant (*Araujia hortorum*)	Also affects *Morrenia odorata, Oxypetalum caeruleum* and *Gomphocarpus* spp.	Ecosystem management	Virus-infected leaves of *Morrenia odorata* in sodium phosphate buffer	Research discontinued because of pathogenicity on *Gomphocarpus fruticosus* Elliott et al. (2009)	Elliott et al. (2009)
Unspecified virus resembling Tobacco Rattle Virus	*Impatiens glandulifera*	Also affects species within *Chenopodium* and *Nicotiana* Kollmann et al. (2007)	Ecosystem management	Virus-infected leaves of *Impatiens glandulifera* in phosphor extraction buffer	Research phase	Kollmann et al. (2007)
\'Obuda Pepper Virus	*Solanum nigrum*	Wide host range Tobias et al. (1982)	Ecosystem management	Mechanical inoculation	Research phase	Kazinczi et al. (2006)
Pepino Mosaic Virus	*Solanum nigrum*	Wide host range including *Amaranthaceae, Chenopodiacee, Compositae, Convolvulaceae, Malvaceae, Plantaginaceae* and *Solanaceae* Papayiannis et al. (2012)	Ecosystem management	Mechanical inoculation	Research phase	Kazinczi et al. (2006)

Within the scientific literature, three genera of fungi have received the majority of attention as bioherbicide candidates (Table 1). In addition to the aforementioned BioMal and Collego, several other species within the genus *Colletotrichum* have been investigated. Additional examples include *C. truncatum*, which has been investigated to control hemp sesbania (*Sesbania exaltata*) (Schisler et al., 1991), and *C. orbiculare*, which was investigated for its potential to control spiny cocklebur (*Xanthium spinosum*) (Auld et al., 1988, 1990). An investigation of the genomes of *C. gloeosporioides* and *C. orbiculare*, found that both species contained a number of candidate genes predicted to be associated with pathogenesis, including plant cell wall degrading enzymes and secreted disease effectors including small secreted proteins (SSPs), the latter of which were shown to be differentially expressed *in planta* according to stage of infection, suggesting that some of these proteins may have specific roles in the infection process (Gan et al., 2013). There is also evidence that both of these *Colletotrichum* species have the ability to produce indole acetic acid (Gan et al., 2013), a plant hormone, derivatives of which are well established herbicide templates (Grossmann, 2010).

Three species within the genus *Phoma* have also received attention as potential agents for biological weed control (Table 1). *P. herbarum*, a fungal pathogen originally isolated from dandelion leaf lesions in Southern Ontario, has been investigated for control of dandelions in turf (Neumann and Boland, 1999; Stewart-Wade and Boland, 2005). *P. macrostoma* has also been investigated for similar purposes as it has been observed to specifically inhibit the growth of dicot plants (Bailey et al., 2011, 2013; Smith et al., 2015). The 94-44B strain of this species has been registered for control of broadleaf weeds in turf systems in Canada and the US (Evans et al., 2013). An investigation of 64 strains of *P. macrostoma*, including 94-44B, found that the bioherbicidal activity of these species was limited to a genetically-homogeneous group of strains, all of which were isolated from Canada thistle (Pitt et al., 2012). Through mass spectrometry, *P. macrostoma* has been recognized to produce photobleaching macrocidins (Graupner et al., 2003) that do not affect monocots (Bailey et al., 2011). As the activity of *P. macrostoma* is most apparent on new growth, it has been suggested that these compounds are transported in the phloem of the host plant (Graupner et al., 2003). Unfortunately, the specific phytotoxic mechanism of macrocidins remains unknown (Schobert and Schlenk, 2008; Zhao et al., 2011; Mo et al., 2014). Despite this, macrocidins and other molecules within the tetramic acid family have received significant attention as templates for the development of novel synthetic herbicides (Barnickel and Schobert, 2010; Yoshinari et al., 2010; Zhao et al., 2011). Additionally, an anthraquinone pigment has been isolated from a *P. macrostoma* strain and shown to have herbicidal effects on several prominent weeds of Central India (Quereshi et al., 2011). Anthraquinone pigments produced by other fungi have also been demonstrated to cause necrosis on wheat leaf blades (Bouras and Strelkov, 2008) and a variety of cultivated legumes (Andolfi et al., 2013). Although the phytotoxic mechanism underlying the effects of these compounds has not been fully characterized, the development of necrosis after exposure to the anthraquinone lentisone was found to be light dependent, a potential clue for the eventual determination of the mechanism associated with this class of molecules (Andolfi et al., 2013). Also of note within this genus is *Phoma chenopodicola*, which has been investigated as a potential control agent for lamb’s quarters (*Chenopodium album*) (Cimmino et al., 2013). A phytotoxic diterpene, chenopodolin, has been isolated from this species, which was found to cause necrotic lesions on lamb’s quarters (*Chenopodium album*), creeping thistle (*Cirsium arvense*), green foxtail (*Setaria viridis*) and annual mercury (*Mercurialis annua*) (Cimmino et al., 2013). Two additional fungal isolates of the genus *Phoma* have also been found to cause a modest degree of stem rot on *C. arvense*, however, these isolates were not identified at the species level (Skipp et al., 2013).

Two species within the aforementioned *Sclerotinia* genus have been investigated for their potential to control weeds. Abu-Dieyeh and Watson (2007a) found that *Sclerotinia minor* effectively controlled dandelions with and without the presence of turf species in greenhouse conditions. A follow up trial including application of *S. minor* in field conditions confirmed these results (Abu-Dieyeh and Watson, 2007b). As noted earlier, *S. minor* strain IMI 344141 was introduced to the Canadian lawn care industry under the product name Sarritor in 2010, however, it is no longer commercially available (Watson and Bailey, 2013; see Challenges in Commercialization). A relative of *S. minor*, *S. sclerotiorum*, has been observed to have phytotoxic activity against creeping thistle (*Cirsium arvense*) (Skipp et al., 2013). Production of oxalic acid by both *S. minor* (Briere et al., 2000) and *S. sclerotiorum* (Magro et al., 1984) has been observed to play a role in the virulence of these fungi on their host plant. Oxalic acid production can be encouraged through addition of sodium succinate to *S. minor* growth media, and cultures grown on sodium succinate-enriched media caused greater development of necrotic tissue when applied to dandelion than cultures grown on non-enriched media (Briere et al., 2000). Oxalic acid acidifies the host tissue, enabling cell wall degradation, and also interferes with polyphenol oxidase (PPO), which normally assists in plant defense (Magro et al., 1984). Low concentrations of oxalic acid have also been shown to suppress the release of hydrogen peroxide, another plant defense molecule, in cell cultures of soy and tobacco (Cessna et al., 2000).

In addition to the other examples described earlier in the text, several other fungi have been registered as bioherbicides for use in forestry or ecosystem management in Canada and the US (Bailey, 2014), though in general, there appears to be limited research about these strains with respect to their mode of action. Two separate strains of *Chondrostereum purpureum* have been registered in Canada and the US for controlling regrowth of deciduous tree species in coniferous plantations (Bailey, 2014). This fungal species is a naturally occurring pathogen of deciduous trees in North America (Setliff, 2002). Although the potential host range of this species is fairly wide, wound infection is a key element of successful infection in most cases (Setliff, 2002). *C. purpureum* strain HQ1 was registered under the product name Mycotech Paste with the PMRA in 2002 (PMRA Reg. No. 27019) and the EPA in 2005 (EPA Reg. No. 74128-2). Registration of this strain with the PMRA ended in 2008. Another strain of this species, PFC 2139, was registered under the product name Chontrol Paste with the EPA in 2004 (EPA Reg. No. 74200-E/R) and with the PMRA in 2007 (PMRA Reg. No. 27823 and 29293). Both registrations are currently active and this product remains commercially available.

Another fungus, *Puccinia thlaspeos*, was registered with the EPA in 2002 under the product name Woad Warrior for control of Dyer’s woad (*Isatis tinctoria*) (EPA Registration Number 73417-1). This fungus is an obligate parasite and requires a living host to reproduce, however, inoculum can be produced from dried and ground plant material of its target weed (Thomson and Kropp, 2004). This product is no longer commercially available (Bailey, 2014).

*Alternaria destruens* strain 059 was registered with the EPA in 2005 under the product names Smolder WP and Smolder G (EPA Reg. Nos. 34704-825 and 34704-824, respectively). This product, originally isolated from *Cuscuta gronovii* growing in unmanaged conditions in Wisconsin, is intended for control of dodder species (*Cuscuta* spp.) (Cook et al., 2009), however, it is not commercially available (Bailey, 2014).

A final bioherbicide that bears mentioning is DeVine, a formulation of the fungus *Phytophthora palmivora* (Kenney, 1986). This product was registered with the EPA in 1981 and again in 2006 (Bailey, 2014; EPA Reg No. 73049-9). *P. palmivora* was originally isolated from strangler vine (*Morrenia odorata*) in Florida and was used to control the same species in citrus orchards (Ridings, 1986). Although this product was re-registered in 2006, it is no longer commercially available (Bailey, 2014).

## Biological Control of Weeds Using Bacteria

A number of bacteria have also been investigated as potential biological weed control agents (Table 1). Of these, *Pseudomonas fluorescens* and *Xanthomonas campestris* have attracted the most attention. Biological weed control using bacteria has been suggested to have several advantages over the use of fungi, including more rapid growth of the bioherbicide agents (Johnson et al., 1996; Li et al., 2003), relatively simple propagation requirements (Li et al., 2003), and high suitability for genetic modification through either mutagenesis or gene transfer (Johnson et al., 1996).

As mentioned above, *P. fluorescens* has received much of the attention as a biological weed control agent (Table 1). There are many strains of this species, some of which are beneficial to plants (Gamalero et al., 2005), whereas others are inhibitory (Banowetz et al., 2008). Among studies into the suppressive effects of *P. fluorescens*, three strains have been investigated in especially great detail, all of which have been observed to inhibit plant growth and/or germination through the production of extracellular metabolites (Kennedy et al., 1991; Quail et al., 2002; Banowetz et al., 2008).

*Pseudomonas fluorescens* strain D7, originally isolated from the rhizospheres of winter wheat (*Triticum aestivum*) and downy brome (*Bromus tectorum*) in Western Canada, has been observed to selectively inhibit growth and germination of a number of grassy weeds, most notably downy brome (Kennedy et al., 1991, 2001; Gealy et al., 1996). By selective removal of compounds from cell-free filtrates, the growth-inhibiting activity associated with this strain was attributed to a combination of extracellular peptides and a lipopolysaccharide, which were suggested to work in conjunction to express herbicidal activity (Gurusiddaiah et al., 1994). No subsequent reports regarding mechanism were found in the available literature.

Conversely, *P. fluorescens* strain WH6 has been observed to affect the germination of a much broader range of plant species, significantly inhibiting germination of all species tested (21 monocot species and 8 dicot species) with the exception of a modern corn (*Zea mays*) hybrid (Banowetz et al., 2008). The germination-inhibiting activity of the WH6 strain has been attributed to the production of a compound originally referred to as Germination Arrest Factor (GAF; Banowetz et al., 2008). The active component of GAF has been identified through nuclear magnetic resonance spectroscopy and mass spectrometry as 4-formylaminooxy-L-vinylglycine (McPhail et al., 2010), and its biosynthesis has been proposed to begin with the amino acid homoserine (Halgren et al., 2013). This class of compounds, the oxyvinylglycines, has been shown to interfere with enzymes that utilize pyridoxal phosphate as a cofactor, including enzymes involved in nitrogen metabolism and biosynthesis of the plant hormone ethylene (Berkowitz et al., 2006; Halgren et al., 2013). Interestingly, GAF has also been recognized to express specific bactericidal activity against *Erwinia amylovora*, the bacterium that causes fire blight in orchards (Halgren et al., 2011). The genome sequence of *P. fluorescens* strain WH6 has been published (Kimbrel et al., 2010), and gene knockouts were used to identify several biosynthetic and regulatory genes involved in GAF/ 4-formylaminooxy-L-vinylglycine production (Halgren et al., 2013; Okrent et al., 2014). Strain D7 was also included in the original investigation of strain WH6, however, as culture filtrates of strain D7 prepared in the same manner as WH6 did not possess germination-inhibiting activity the authors suggested that GAF was not responsible for the activity associated with strain D7 (Banowetz et al., 2008).

The production of extracellular metabolites with phytotoxic effects has also been observed in an additional *P. fluorescens* strain, referred to as BRG100, which has been recognized to have suppressive activity on the grassy weed green foxtail (*Setaria viridis*) (Quail et al., 2002; Caldwell et al., 2012). The herbicidal compounds produced by this species, referred to as pseudophomin A and B, have been characterized through a combination of serial chromatography, high performance liquid chromatography (HPLC), thin layer chromatography (TLC), chemical degradation, and X-ray crystallography (Quail et al., 2002; Pedras et al., 2003). Unfortunately, neither the biosynthetic pathway involved in the production of these compounds nor the specific biochemical effects of these molecules on green foxtail have been characterized at this time. However, the full genome sequence of this strain has been published (Dumonceaux et al., 2014) and a detailed projection of the costs and technical requirements for the mass production of this biocontrol agent has been reported (Mupondwa et al., 2015).

The other bacterial species that has received much of the attention as a candidate biological weed control agent is *Xanthomonas campestris* (Table 1). Most notably within this species, the strain *X. campestris* pv. *poae* (JT-P482) was registered in Japan in 1997 for control of annual bluegrass (*Poa annua*) under the product name Camperico (Imaizumi et al., 1997; Tateno, 2000). The activity of this species is specific to *Poa annua* and *Poa attenuata*, and was not reported to affect other turf species tested (Imaizumi et al., 1997). A separate strain of *X. campestris* (isolate LVA-987) has also received attention as a potential control agent against horseweed (*Conyza canadensis*) (Boyette and Hoagland, 2015). No discovery of phytotoxic compounds was reported in any of the aforementioned investigations into application of *X. campestris* as a bioherbicide, however, compounds with phytotoxic activity have been previously isolated from the *vitians* pathovar of this species (Scala et al., 1996), and it is possible that phytotoxic metabolites play a role in the suppression of *Poa annua* and *Conyza canadensis*. Although the cause of host-plant suppression was not indicated in the above studies, the infection process of *X. campestris* pv. *campestris* (*Xcc*) in brassica crops has been well characterized. Briefly, *Xcc* can colonize the xylem of the host plant and use this pathway to spread throughout the organism (Duge de Bernonville et al., 2014). The success of *Xcc* in reaching the host xylem is contingent on its interaction with receptor proteins of the host plant that can recognize pathogen associated molecular patterns (PAMPs), potentially resulting in elicitation of plant defense responses such as programmed cell death and increased production of reactive oxygen (Guy et al., 2013).

## Biological Control of Weeds Using Viruses

In select cases, viruses that affect weed species have also been considered as bioherbicide candidates. This strategy is more commonly considered for management of invasive species in broader ecosystems rather than specifically managed areas. Viruses have been suggested to be inappropriate candidates for inundative biological control due to their genetic variability and lack of host specificity (Kazinczi et al., 2006). Examples of viruses that have been investigated for the potential to control invasive or undesirable species include Tobacco Mild Green Mosaic Tobamovirus for control of tropical soda apple (*Solanum viarum*) in Florida (Ferrell et al., 2008; Diaz et al., 2014), and *Araujia* Mosaic Virus for control of moth plant (*Araujia hortorum*) in New Zealand (Elliott et al., 2009). A patent on the former biological control agent has been filed (Charudattan et al., 2009) and EPA approval for use on fenced-in pasture areas was granted in 2015 (EPA, 2015). A virus resembling Tobacco Rattle Virus has also been proposed as a control agent for *Impatiens glandulifera*, an invasive weed of concern in central and western Europe (Kollmann et al., 2007). Similarly, Óbuda Pepper Virus (ObPV) and Pepino Mosaic Virus (PepMV) have been proposed as agents to reduce overall populations of the weed *Solanum nigrum* (Kazinczi et al., 2006). The biological activities of viruses are very distinct from pathogenesis caused by bacteria or fungi, and may present additional opportunities for biological weed control in some situations.

## Real World Factors that Affect the Efficacy of Bioherbicides

**FIGURE 1:**
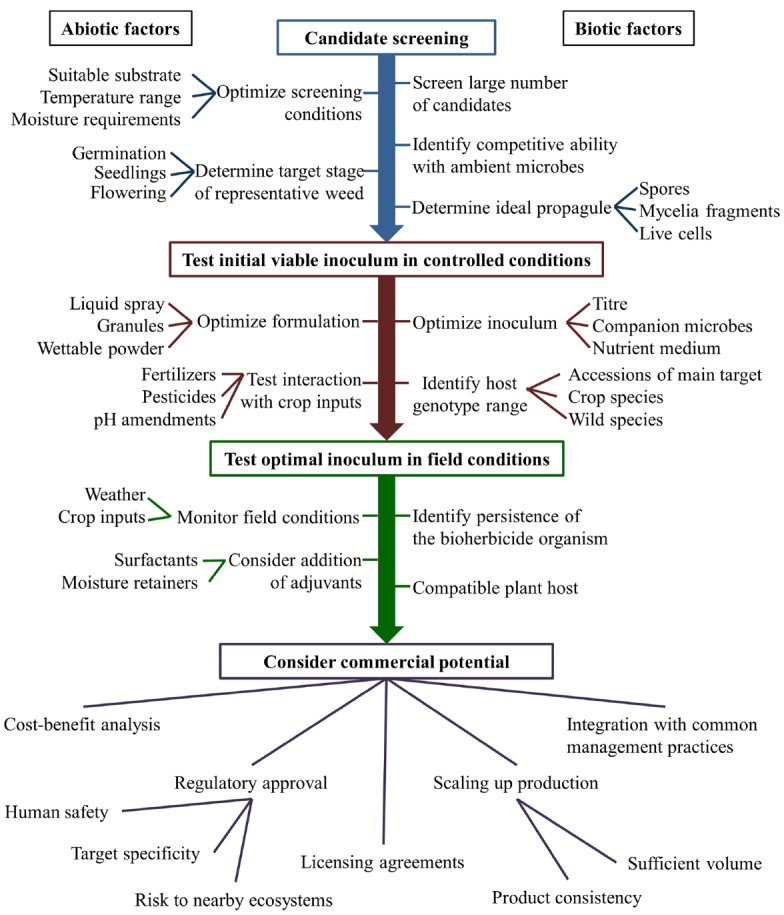
**Overview of factors involved in the development of a bioherbicide**.

The research pipeline from the screening stage to field conditions faces a number of unique challenges (Figure 1). One commonly reported challenge is the need for continuous moisture availability during the period in which the biocontrol agent infects the plant (Auld et al., 1990; Schisler et al., 1991; Auld and Morin, 1995; Stewart-Wade and Boland, 2005; Boyette and Hoagland, 2015). In a review of bioherbicide technology published by Auld and Morin (1995), it was reported that dew periods of more than 12 hours are commonly necessary for bioherbicide candidates to successfully infect their hosts. A variety of techniques to provide this moisture have been tested, with varying degrees of success. In order to prolong the period of leaf wetness necessary for successful infection of dandelion by *Phoma herbarum*, several vegetable oil emulsions were included in aqueous inoculants, however, these additives were found to be phytotoxic, thus obscuring the benefit to infection that may have been caused by their addition (Stewart-Wade and Boland, 2005). Timing inoculant application to prolong the leaf wetness period (e.g., application at dawn or dusk) has also been suggested as a simple method of maximizing infection, although the success of this technique can be highly sensitive to environmental fluctuations (Auld and Morin, 1995). In some cases, solid inoculant media have also been investigated. The most common method for developing solid inoculant media is to propagate the candidate biological weed control species on grains which will subsequently be applied directly to the field or incorporated with other moisture-retaining materials such as calcium alginate, oils or vermiculite (Auld et al., 2003). Granular applications have the advantage of prolonging the in-field survival of introduced biological weed control agents through the provision of moisture and nutrients, however, they are also generally associated with a more gradual rate of infection (Auld et al., 2003).

The interplay of temperature and humidity also has a significant effect on the success or failure of infection by many pathogens (Ghosheh, 2005) and may alter the efficacy of biological control agents (Casella et al., 2010; Figure 1). Cold air can retain less total moisture than warm air, and thus the relative humidity is more commonly elevated at lower temperatures. Elevated humidity is generally beneficial to successful bioherbicide colonization because it decreases evaporation rates, thus increasing the duration of leaf wetness following inoculant application (Casella et al., 2010). In investigating the efficacy of the biological weed control product Sarritor, it was found that infection rates were highest when the temperature remained below 20°C and the relative humidity was high (Siva, 2014). Similar requirements have been suggested by Auld et al. (1990), who observed that successful infection of spiny cocklebur (*Xanthium spinosum*) by biological weed control agent *Colletotrichum orbiculare* may have been contingent on elevated humidity. As with any species, most biological control species will have a fairly finite temperature range in which they can survive, as well as a narrower range in which their activity will be maximized. For example, Imaizumi et al. (1997) found that ambient temperatures below 25°C (day) and 20°C (night) caused decreased efficacy of *Xanthomonas campestris* pv. *poae* in the suppression of annual bluegrass (*Poa annua*). Similarly, the efficacy of *X. campestris* isolate LVA-987 in controlling horseweed (*Conyza canadensis*) was found to require ambient temperatures between 20°C and 35°C, with peak efficacy between 25°C and 35°C (Boyette and Hoagland, 2015). This parameter will be different for any given biological control candidate species and thus temperature and humidity should be tracked throughout any efficacy trials involving biological weed control agents.

Quorum sensing refers to the ability of a bacterium to differentially express genes based on its population density (Rutherford and Bassler, 2012). The effect of bacterial and fungal population densities can in some cases inform the behavior of these species, and in some cases affect whether a pathogen is virulent or latent (Bowden et al., 2013; Lu et al., 2014). This is an important factor in the characterization of potential bioherbicides, however, testing inoculant media with varying population densities (Figure 1) is not a common practice within the investigation of biological weed control strategies, nor is the phenomenon of quorum sensing commonly discussed in the related literature. However, apparent latent periods in the life cycle of biological weed control candidate species have been occasionally reported (Romero et al., 2001; Paynter et al., 2006), and it is possible that quorum sensing effects could explain these cases of asymptomatic infection.

It is possible that interactions with fertilizers and pesticides could affect the infectiousness of a candidate biological weed control agent (Boyetchko, 1997; Figure 1). For example, an investigation of the ability of *P. macrostoma* to control dandelions in turf found that co-application with a high rate of nitrogen fertilizer improved its efficacy, whereas co-application with phosphorus had no effect, and potassium sulfate decreased efficacy (Bailey et al., 2013).

## Challenges in Commercialization

Despite the promise shown by many bioherbicides, few have achieved long-term commercial success, in part due to the challenges to achieving consistent efficacy in field conditions noted above. For example, amongst the fungal bioherbicides described in this review, only LockDown (*C. gloeosporioides* f.sp. *aeschynomene*) remains commercially available (Bailey, 2014). In the case of BioMal (*C. gloeosporioides* f.sp. *malvae*), the narrow target specificity (only round leaf mallow) of the product made for a market niche that was too small to cover production costs (Cross and Polonenko, 1996). Additionally, significant challenges were encountered in maintaining product consistency while scaling up production volumes (Boyetchko et al., 2007). For these reasons, Philom Bios, the original commercial producer of this bioherbicide, discontinued its production in 1994, only 2 years after registration (Boyetchko et al., 2007). The strain was later licensed to Encore Technologies in 1998, however, challenges with maintaining product consistency under mass production led to the abandonment of the project (Boyetchko et al., 2007). In the case of Sarritor (*S. minor* strain IMI 344141), the commercial failure has been attributed to challenges with increasing production volume and product consistency, as well as inconsistent efficacy of the product due to the narrow range of environmental conditions in which successful infection will occur (Watson and Bailey, 2013). Unfortunately, the current commercial status of Camperico (*X. campestris* pv. *poae* JT-P482), described above, is unclear (Bailey, 2014).

## Future Directions

### Mechanism of Action

As noted above, the mechanism(s) behind the suppressive activity of a given biocontrol agent is in many cases only partially understood. Future research into the mechanisms underlying these effects will be important to achieve consistent efficacy with biocontrol agents, as well as to evaluate potential impacts on human and ecosystem health. This in turn will be of value to gaining regulatory approval. Additionally, understanding bioherbicidal mechanisms may generate novel chemical herbicides to overcome current resistance traits (Boyetchko et al., 2009), and will likely also be of peripheral value to the field of plant pathology.

### Transition to the Field

Translating effects observed in a controlled environment to field conditions is a significant challenge to the development of successful biocontrol agents (Figure 1), and it is common for projects to conclude at this juncture. Thus, the development of new delivery formulations intended to improve the in-field stability of biocontrol agents is as important as the discovery of the agents themselves. Widespread testing of a given biocontrol agent in a variety of locations, similar to plant variety testing, is essential to understanding the feasibility of introducing that agent on a broad scale. Finally, the production of commercially relevant quantities of viable inoculum or culture extract must also be considered, as techniques employed in the laboratory are frequently impractical for industrial-scale production. Lessons from other industries such as pharmaceuticals and probiotic foods will likely be valuable in addressing this challenge.

### Extraction of Herbicidal Compounds

As discussed earlier, in some cases a particular herbicidal compound can be extracted from a live culture (Kennedy et al., 1991; Quail et al., 2002; Banowetz et al., 2008). This strategy can yield a more stable control agent, the efficacy of which will not be contingent on the continued survival of a given organism in an uncontrolled environment. Although the differentiation between naturally and synthetically sourced pesticides may be arbitrary in terms of their potential effect on human and environmental health, such compounds will likely be more acceptable in the public eye than those produced through traditional chemistry.

### New Sources of Bioherbicide Candidates

This review has focused on a limited number of genera which have received an especially great degree of attention as bioherbicide candidates for turf and field crop situations. Considering the degree of taxonomic diversity among microbes, there are opportunities to employ other genera as bioherbicides in the future. Most of the studies discussed in this review employed microbes that were originally isolated from diseased individuals within the population of a weed species (Kennedy et al., 1991; Neumann and Boland, 1999; Ghosheh, 2005). However, there are additional ecological niches from which potential biological weed control candidates can be discovered. For example, most plant species form relationships with a variety of microbes, referred to as endophytes, which colonize the internal environment of the plant without causing disease (Duan et al., 2013; Ali et al., 2014). There is evidence that endophytes can play a role in nutrient accumulation, drought tolerance and disease resistance (Compant et al., 2010; Johnston-Monje and Raizada, 2011; Mousa and Raizada, 2013). Growth-promoting endophytes have been shown to reduce weed populations in pastures by inoculating the desired grass species, enabling them to compete with weeds more effectively (Saikkonen et al., 2013; Vazquez-de-Aldana et al., 2013). It has been reported that some plant-inhabiting microbes will express host-specific behavior, acting as an endophyte in some plant species but as a pathogen in another (Gomes et al., 2013). Additionally, some endophytes have also been reported to produce compounds that are phytotoxic to non-host species (Waqas et al., 2013; Zhang et al., 2013; Li et al., 2014). These phenomena could potentially be applied to controlling undesirable weed species. Endophyte-based weed control may have unique advantages over the application of pathogens such as improved ability of candidate microbes to persist in field conditions through having a more consistent ecological niche within their plant host, or the provision of other benefits to their host such as nutrient acquisition or disease resistance.

## Conclusion

Although there are many challenges and constraints inherent in the development of biological herbicides, the increasing prevalence of both herbicide-resistant weeds (Green and Owen, 2011) and public concern with pesticide use (McNeil et al., 2010) creates a strong impetus for continued investigation in this field. These strategies will be of especially great value to organic production systems and to regions where cosmetic pesticide bans are in place. With continued investigation in this field, there is significant potential for the development of new weed control strategies that can be employed to delay herbicide resistance, produce food in accordance with consumer concerns, and reduce the environmental impact of modern agriculture and ecosystem management. Although there is a considerable number of candidate species that have been considered for this purpose, the major challenge to successful implementation of this strategy is the development of techniques to maintain consistent efficacy in field conditions.

## Author Contributions

DPH conceived and wrote the manuscript, and MNR edited the manuscript. Both authors approved the final manuscript.

### Conflict of Interest Statement

The authors declare that the research was conducted in the absence of any commercial or financial relationships that could be construed as a potential conflict of interest.
